# Two Children With Steroid-Resistant Significant Proteinuria Due to Nonsense Mutations of the *TRIM8* Gene: A Case Report and Literature Review

**DOI:** 10.3389/fped.2022.918373

**Published:** 2022-07-12

**Authors:** Xiaojie Li, Yaqin Wei, Meiqiu Wang, Lili Jia, Zhuo Shi, Xiao Yang, Tao Ju, Qianhuining Kuang, Zhengkun Xia, Chunlin Gao

**Affiliations:** ^1^Affiliated Jinling Hospital, Medical School of Nanjing University, Nanjing, China; ^2^Department of Pediatrics, Jinling Hospital, The First School of Clinical Medicine, Southern Medical University, Nanjing, China; ^3^Department of Pediatrics, Jinling Hospital, Nanjing, China; ^4^Department of Pediatrics, Jinling Hospital, Nanjing Medical University, Nanjing, China

**Keywords:** *TRIM8* gene, steroid-resistant significant proteinuria, FSGS, epileptic encephalopathy, children

## Abstract

**Background:**

*TRIM8* gene mutations have been reported as the genetic basis of autosomal dominant (AD) neuro-renal syndrome in children, which presents with epileptic encephalopathy, focal segmental glomerulosclerosis (FSGS), developmental delay, and mental retardation. In this study, we report the cases of two children with significant proteinuria due to de novo nonsense mutations of the *TRIM8* gene.

**Case Presentation:**

Case 1 was a 7-year-old girl who presented with proteinuria and developmental delay, and her renal biopsy showed FSGS. She developed end-stage renal disease (ESRD) 3 years after onset. Case 2 was another 7-year-old girl who developed proteinuria only at age 3, and renal biopsy showed glomerular segmental mesangial proliferative lesions. The two girls underwent genetic testing but we did not find a positive result in the whole exon. However, cluster analysis revealed two new nonsense mutations of the *TRIM8* gene (c.1461C>A, p.Tyr 487^*^ and c.1453C>T, p.Gln485^*^).

**Conclusions:**

We reported the clinical manifestation of this neuro-renal syndrome for the first time in China. It is necessary to perform genetic testing in children with steroid-resistant significant proteinuria to identify its etiology and avoid the side effects of immunosuppressants.

## Introduction

With the popularization of gene sequencing technology, the detection rate of hereditary nephropathy in children is increasing each year, and more information is now available to aid in the human understanding of diseases. Hereditary nephropathy may make children vulnerable to steroid-resistant or multiple immunosuppressant-resistant nephroses. We reported two children with steroid-resistant significant proteinuria and did not find positive results in the whole exome sequencing. However, cluster analysis revealed two new mutations in *TRIM8* exon 6. Recent studies have suggested that the mutation of the *TRIM8* gene is the genetic basis for the early onset of neuro-renal syndrome (1, 2).

## Case Presentation

### Case 1

**Figure 1:**
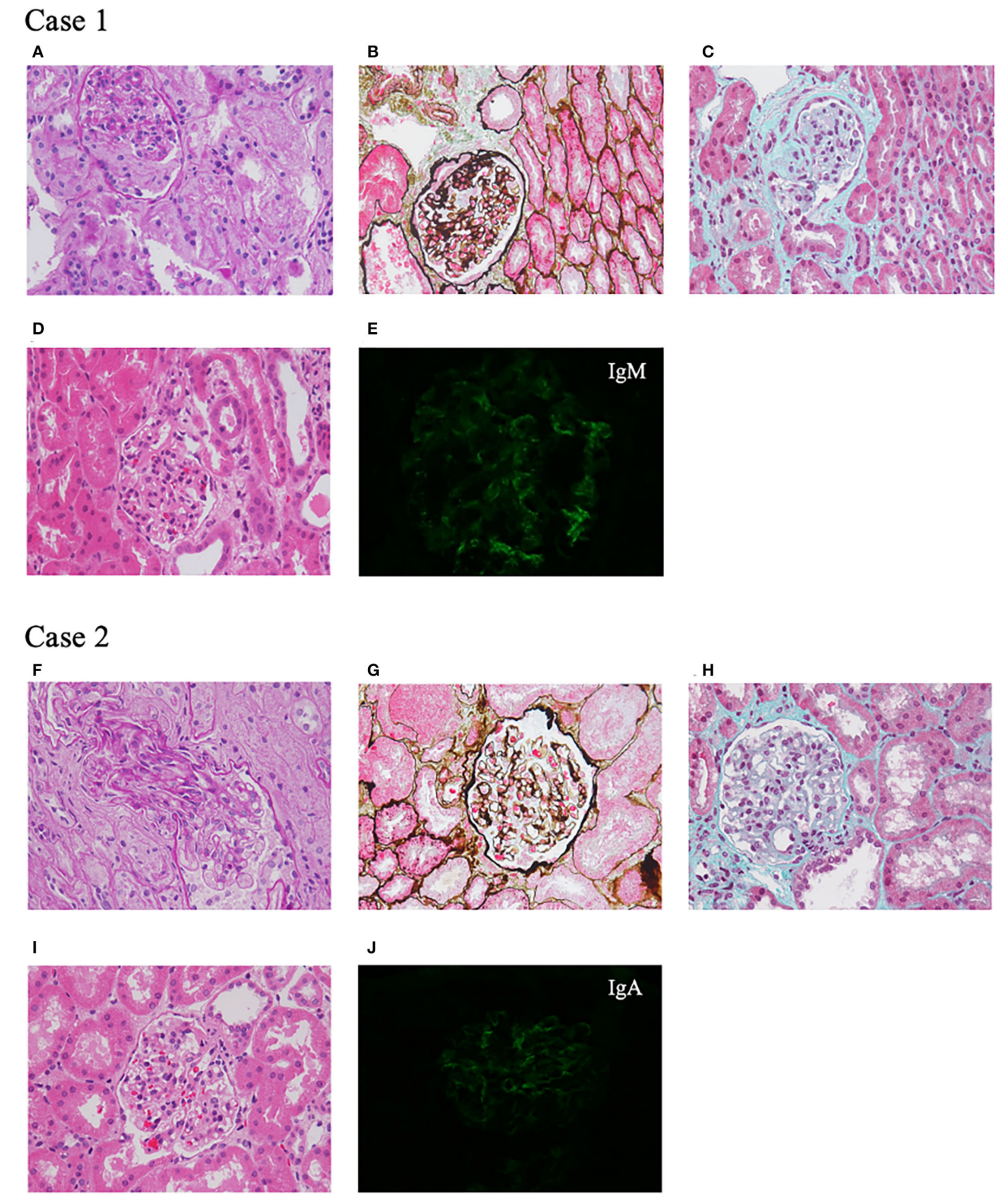
Renal pathological presentation of two probands. **(A–D,F–I)** Light microscopy. **(A,F)** Periodic Acid Schiff staining, ×400 magnification. **(B,G)** Periodic acid-silver methenamine staining, ×400 magnification. **(C,H)** Masson staining, ×400 magnification. **(D,I)** Hematoxylin-eosin staining, ×400 magnification. **(E,J)** Immunofluorescence.

A 7-year-old girl, born at full term to unrelated healthy Chinese parents with no family history of renal disease or epilepsy, showed a mild developmental delay in walking at 18 months without language retardation. At 3 years and 1 month, she had mild periorbital and bilateral lower limb edema. She was average in height and weight for her age, without special facial features. Her physical examination showed that her heart and nervous system were normal. Urine tests showed mild hematuria (62.9 red blood cells/HPF) and nephrotic range proteinuria (24-h proteinuria: 1.007–1.820 g). The blood biochemistry was normal except for a slightly low albumin level of 36.1 g/L and a mildly high total cholesterol level of 5.87 mmol/L. Other laboratory results, such as levels of anti-nuclear antibodies, anti-double stranded DNA antibodies, hepatitis B virus, and human immunodeficiency virus were negative, and renal ultrasound showed no obvious abnormalities. At 3 years and 10 months of age, her percutaneous renal biopsy demonstrated segmental glomerular sclerosis (1/10) categorized as global obsolete (2/10), and immunofluorescence staining showed IgM trace, which suggested focal segmentary glomerulosclerosis (FSGS) (Figure 1). Her treatment included 4 weeks of 1.5–2 mg/kg prednisone followed by 0.1–0.15 mg/kg tacrolimus, but she did not respond to these drugs. Later, her renal function declined, and she began peritoneal dialysis at 6 years of age. The child had convulsions twice when she was 7 years old, but the electroencephalogram and MRI of the brain were normal. Considering the convulsions were caused by hypertension, her treatment included anticonvulsants and antihypertensive drugs. No convulsions have occurred since then.

### Case 2

A 7-year-old Chinese girl was born at full term. Her father was healthy, while her mother was diagnosed with FSGS and renal stones, and her grandfather also had renal stones. After she was born, there were no signs of motor or language retardation. She had proteinuria and microscopic hematuria during a routine examination at the age of 3 years and 4 months. Her physical tests were negative including heart, lung, liver, and nervous system examinations. The urine dipstick test was 1+-3+ positive for protein with 24–84 red blood cells/HPF, and 24-h protein excretion was 1.7 g. The levels of serum creatinine, albumin, anti-double stranded DNA antibodies, anti-nuclear antibodies, and complements were all within normal limits. At 3 years and 5 months of age, renal ultrasound showed a hypoechoic cortex and no renal stones. Renal biopsy revealed glomerular segmental mesangial proliferative lesions, and immunofluorescence staining was IgA+ (Figure 1). After treatment with 1.5–2 mg/kg prednisone, her proteinuria decreased but was not in complete remission. Tacrolimus and cyclophosphamide were added to steroids at different times but did not achieve remission. During the 4 years of follow-up, her urine protein was maintained at 0.3–0.6 g/24 h with normal renal function.

**Figure 2:**
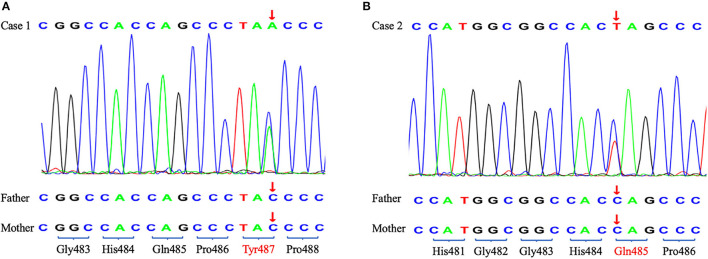
The Sanger sequencing of two probands and their parents. The arrow shows the position of the *de novo* nonsense mutations. **(A)** Case 1. **(B)** Case 2.

To identify the pathogeny, the two girls and their families underwent whole exome sequencing (WES) in 2019 but we did not find an obvious pathogenic mutation. WES covers the coding sequence of nearly 20,000 functional genes containing all exons of *TRIM8*. The genome coverage and the average depth of coverage were 99% and 134.89 × in case 1 and 99% and 132.66 × in case 2, respectively. However, the cluster analysis in 2021 found two *de novo* nonsense mutations in exon 6 of the *TRIM8* gene. Case 1 showed c.1461C>A, p.Tyr487^*^ and case 2 showed c.1453 C>T, p.Gln485^*^ (Figure 2). According to the American College of Medical Genetics and Genomics (ACMG) guidelines (2019), the variants were also pathogenic.

## Discussion

Cluster analysis is a mathematical-statistical method to classify research subjects according to the information provided by the research subject, which is used widely in genetic testing datasets (3). It is a process of finding commonalities, in which a gene is run through a large phenotypic-genotype database to see if patients with suspected mutations in that gene have similar phenotypes. The cluster system used in this study included clinical and genetic test data from more than 100,000 Chinese patients. We first noticed that case 1 carried a *de novo* LOF (loss of function) mutation of *TRIM8*, and then we found that *TRIM8* is highly expressed in the renal according to the Genotype-Tissue Expression (GTEx) database, which suggests that it may be related to renal disease. Therefore, we searched our database and found that these two girls with mutations in exon 6 of the *TRIM8* gene had similar phenotypes.

In 2013, the Epi4K project, which aimed to decipher the genetic causes of epilepsy by WES, found a *de novo* frameshift mutation in the *TRIM8* gene (4). In 2018, Mirna Assoum reported another four epileptic encephalopathy patients with *TRIM8* variants (2). In 2021, Weng et al. discovered a nonsense variant in *TRIM8* in a child who presented with epilepsy, developmental delay, and SRNS/FSGS (5). Then, they detected eight heterozygous *TRIM8* truncating variants from 2,501 pediatric FSGS/SRNS-affected individuals and three *TRIM8* variants from 9,067 individuals with epilepsy. To date, a total of 19 children with 15 mutations in exon 6 of the *TRIM8* gene have been found, except for the two reported in this paper (Table 1). The 19 patients had a similar ratio of males to females, with ten males and nine females. Eighty percent of patients (15/19) presented with nephrotic-range proteinuria, and fifty-eight percent (11/19) of biopsies showed FSGS. Sixty-eight percent of them (13/19) developed ESRD, and only six children received renal transplantation. All patients had developmental delays, and only one boy did not develop epilepsy.

**Table 1:** Mutations in exon 6 of the *TRIM8* gene have been reported.

**Nucleotide change**	**Amino acid change**	**Gender**	**Ethnicity**	**Renal disease**	**Renal disease onset (y)**	**ESRD onset (y)**	**DD onset (y)**	**Epilepsy onset (y)**	**References**
c.1338T>A	p.Tyr446*	F	ND	SRNS; Bx MsGN	4	None	1.3	2	(2)
c.1117_1117del	p.Ala374Argfs*16	M	ND	None	None	None	0.5	0.5	(2)
c.1331C>A	p.Ser444*	F	ND	NRP	ND	None	<0.5	3.4	(2)
c.1267C>T;	p.Gln423*	M	ND	None	None	None	0.4	1.7	(2)
		M	African American	NS; Bx FSGS; Tx	11	12	0.5	2	(5)
c.1375C>T	p.Gln459*	F	ND	Proteinuria	0.6	None	0.4	1.7	(2)
		M	Korean	SRNS; Bx FSGS; Tx	4	5	<1	2	(5)
		M	German	NRP; Bx FSGS	7.9	9.8	2.5	2.5	(5)
		F	European	NS; Bx FSGS	3	5	<1	2.5	(5)
c.1099_1100insG	p.Cys367Trpfs*43	M	Japanese	None	None	None	0.4	0.2	(6)
c.1163delT	p.Phe388Serfs*	F	European	SRNS; Bx FSGS; Tx	2.2	3	2.2	4.5	(5)
c.1231C>T	p.Gln411*	M	Turkish	SRNS; Bx FSGS; Tx	4.5	4.8	1	2.5	(5)
c.1240C>T	p.Gln414*	F	German	SRNS; Bx DMS; Tx	Birth	1.1	<1	2	(5)
c.1333C>T	p.Gln445*	F	Italian	SRNS; Bx FSGS	13.7	19.7	1.5	1.5	(5)
c.1380T>G	p.Tyr460*	F	Middle eastern	NRP; Bx FSGS	6	8	0.5	1.5	(5)
c.1380T>A	p.Tyr460*	M	Hispanic	SRNS; Bx FSGS;	2.5	>5	<1	2.5	(5)
c.1461C>G	p.Tyr487*	M	Irish/Hispanic	SRNS; Bx FSGS; Tx	6	14	1.5	None	(5)
c.1201_1202 delGGInsTA	p.Gly401*	F	European/South Asian	SRNS; Bx DMS	3	5	1	3	(5)
c.1198_1220del	p.Tyr400ArgfsTer2	M	British	NRP; Bx FSGS	2.1	5	<1	5	(7)

*Bx, biopsy; DD, developmental delay; DMS, diffuse mesangial sclerosis; ESRD, end-stage renal disease; F, female; FSGS, focal segmental glomerulosclerosis; M, male; MsGN, mesangial glomerulonephritis with non-specific IgM deposits; ND, no data; NRP, nephrotic-range proteinuria; NS, nephrotic syndrome; SRNS, steroid-resistant nephrotic syndrome; Tx, transplant*.

**Figure 3:**
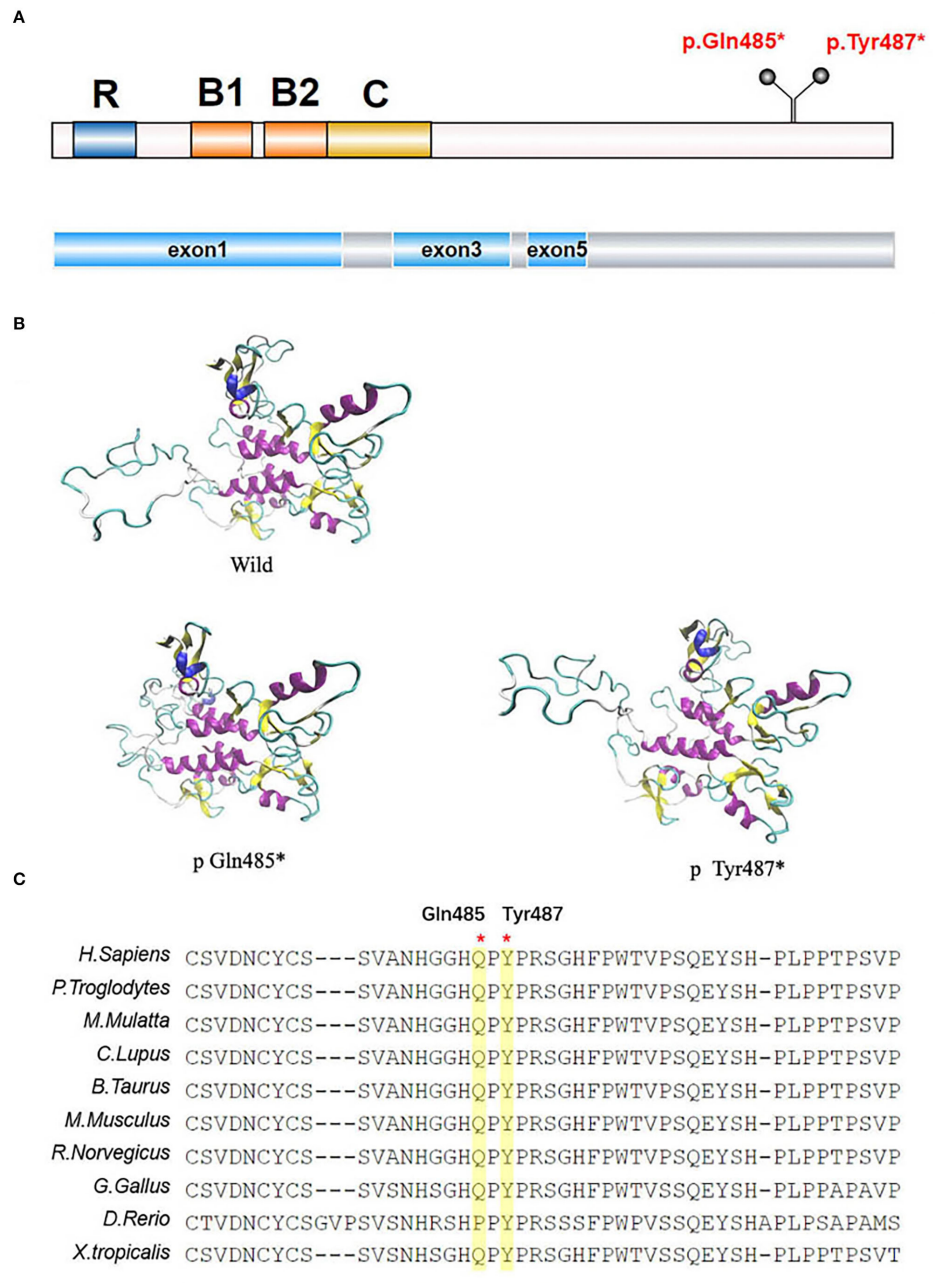
*TRIM8* gene mutations. **(A)** The location of the two *de novo* nonsense *TRIM8* mutations. TRIM8 contains a RING domain at the N-terminal, two B-box motifs (B1 and B2), a Coiled-Coil domain, a Nuclear Localization Signal (NLS) and an RFL-like domain at the C-terminal. **(B)** Structural modeling of *TRIM8* mutations. **(C)** Multiple sequence alignment showing the conservation of the two amino acids between vertebrates.

The *TRIM8* gene is located on chromosome 10q24.3 and encodes a protein of 551 amino acids long with a molecular weight of 61.5 κDa (8). The TRIM8 protein belongs to the TRIM (TRIpartite Motif-containing)/RBCC family, which has more than 70 members, representing one of the largest groups of the E3 ligase RING (really interesting new gene) family in humans. It contains a RING domain at the N-terminal, two B-box motifs (B1 and B2), a Coiled-Coil domain, a Nuclear Localization Signal (NLS), and an RFL-like domain at the C-terminal (Figure 3) (8, 9). TRIM proteins are extensively involved in regulating cell functions, including proliferation, differentiation, and signal transduction (8), and in many biological processes, such as ubiquitination modification. It has been reported that the 15 mutations in exon 6 of the *TRIM8* gene lie between amino acids 367–487. This may indicate that neuro-renal syndrome is associated with the loss of the C-terminal. According to research findings, the C-terminal of the TRIM8 protein is highly conserved in vertebrates. The *TRIM8* gene is highly expressed in the kidney, intestinal tract, and central nervous tissue, which can also explain how the loss-of-function mutations of the *TRIM8* gene cause this disease phenotype.

The two mutations we reported in this study were located in amino acids 485/487, including the truncated mutations in exon 6 that were at the C-terminal of the TRIM8 protein. According to structural analysis, TRIM8 conducts homodimerization through its coiled helical- structure, and its C-terminal is necessary for correct nuclear localization. The mutation leading to translation termination is located after the NLS, causing the sequence length to shorten. This shortened sequence may be detrimental to the stability of nuclear localization. Weng et al. found that the mutated TRIM8 protein was distributed diffusely in the nucleoplasm and could not be limited to the nuclear bodies (5). Warren et al. found low TRIM8 expression and strong suppressor of cytokine signaling 1(SOCS1) expression in the renal biopsy tissues of Hispanic male children (1). Typically, TRIM8, as an E3 ubiquitin-protein ligase, promotes the proteasomal degradation of SOCS1 to participate in the activation of interferon-γ signaling (10). However, this function is impaired in *TRIM8* mutations, and the specific mechanism needs further study.

Compared with previously reported cases, only case 1 of these two patients had significant motor retardation, normal language development and intelligence, and no seizures. However, both cases showed a renal phenotype presenting with nephrotic-range proteinuria. Renal biopsy in case 1 showed FSGS, progression to ESRD within 3 years, and initiation of peritoneal dialysis. This conformed to the characteristics of the previously reported cases. Case 2 had mild symptoms but did not respond to steroids, and a variety of immunosuppressive therapies could not achieve a complete remission or maintain remission. She maintained 0.3–0.6 g/24 h proteinuria with normal renal function, indicating that patients without a neural phenotype may have a better prognosis, but further research is needed to verify this hypothesis. According to the previous studies, most patients (13/19) progressed to ESRD, and 6 of them received renal transplantation. None of the five patients with 24-year follow-up data had clinical recurrence (5). This showed that renal transplantation might be an option for patients with ESRD.

## Conclusion

In conclusion, a neuro-renal syndrome caused by *TRIM8* gene mutation is a rare childhood-onset AD disease representing significant proteinuria and FSGS accompanied by epilepsy and developmental delay. Most patients have a poor prognosis, so early diagnosis and treatment are necessary. Genetic testing and cluster analysis are helpful in finding patients with atypical phenotypes.

## Data Availability Statement

The original contributions presented in the study are included in the article/supplementary material, further inquiries can be directed to the corresponding author/s.

## Ethics Statement

The studies involving human participants were reviewed and approved by Clinical Trials Ethics Committee of the Chinese People's Liberation Army General Hospital of Eastern Theater Command. Written informed consent to participate in this study was provided by the participants' legal guardian/next of kin. Written informed consent was obtained from the minor(s)' legal guardian/next of kin for the publication of any potentially identifiable images or data included in this article.

## Author Contributions

XL, YW, MW, LJ, ZS, XY, TJ, QK, ZX, and CG drafted the manuscript or revised it critically for important intellectual content, provided final approval of the version to be published, and agreed to be accountable for all aspects of the work in ensuring that questions related to the accuracy or integrity of any part of the work were appropriately investigated and resolved. All authors contributed to the article and approved the submitted version.

## Funding

Funding was provided by the Pediatric Medical Innovation Team of Jiangsu Province (CXTDA2017022).

## Conflict of Interest

The authors declare that the research was conducted in the absence of any commercial or financial relationships that could be construed as a potential conflict of interest.

## Publisher's Note

All claims expressed in this article are solely those of the authors and do not necessarily represent those of their affiliated organizations, or those of the publisher, the editors and the reviewers. Any product that may be evaluated in this article, or claim that may be made by its manufacturer, is not guaranteed or endorsed by the publisher.
